# Stress among First and Third Year Medical Students at University Kebangsaan Malaysia

**DOI:** 10.12669/pjms.311.6473

**Published:** 2015

**Authors:** Abdus Salam, Raynuha Mahadevan, Amir Abdul Rahman, Norsyafiqah Abdullah, Aimi Aqilah Abd Harith, Chu Pei Shan

**Affiliations:** 1 Abdus Salam, MBBS; MPH; PgDipMedEd, MMedEd. Associate Professor, Medical Education Department,; 2 Raynuha Mahadevan, BA Hons; MA. Lecturer and Clinical Psychologist, Department of Psychiatry,; 3 Amir Abdul Rahman, Final Year Medical Students, Medical Education Department, Special Study Module Research Group-47, Session 2013/2014,; 4 Norsyafiqah Abdullah, Final Year Medical Students, Medical Education Department, Special Study Module Research Group-47, Session 2013/2014,; 5 Aimi Aqilah Abd Harith, Final Year Medical Students, Medical Education Department, Special Study Module Research Group-47, Session 2013/2014,; 6 Chu Pei Shan, Final Year Medical Students, Medical Education Department, Special Study Module Research Group-47, Session 2013/2014,

**Keywords:** Coping strategies, Medical students, Stress prevalence

## Abstract

**Objective:**

To identify the stress-prevalence and coping-strategies among University Kebangsaan Malaysia (UKM) medical students.

**Methods:**

This was an observational study conducted among 234 UKM first and third year medical students. Standardized questionnaire on stress and coping strategies was used. Stress data was related to subjective experiences on some positive and negative adjectives such as tense, relaxed etc. Positive adjectives were measured by sign “++” and “+” scoring “1” while stress-negative adjectives were measured by sign “?” and “–“ scoring “0”. Forty-eight coping items under task, emotion and avoidance strategies were measured using 5-point Likert-scale.

**Results:**

Overall stress-prevalence was 49%. Female and Malay respondents were more stressed. Significant differences of stress-level was observed between Malays and non Malays in first year (p=0.04) and in third year (p=0.01). Most common strategies used to cope stress was task-oriented while emotion oriented was least.

**Conclusion:**

Stress-prevalence and stress-level in UKM medical students was high. Most of the respondents coped stress using task-oriented strategies. Stressor and its effective management must be ensured. Educational institutions should act as a creative designer of learning environment to get relieve from educational stressor.

## INTRODUCTION

Stress is a condition which upsets an individual both mentally and physically, results from the dealings of the individual with the environment and perceived as threat to the well-being of the individual.^1^ It is well documented that higher education is very stressful and medical education is even more stressful as compared to other professional students.^1^^,^^2^ Study reported that 56% of Malaysian medical students are stressed^1^ and the transition from pre-clinical to clinical year is a crucial stage for level or severity of stress^3^ as the students are more prone to become exhausted due to new environment when entering clinical year. Stress among Universiti Kebangsaan Malaysia (UKM) medical students was found 50% compared to students of Universiti Malaysia Sabah and Royal College of Medicine Perak, which were 42% and 43% respectively.^4^ There is greater risk of stress in women compared to men.^5^ Malay students have significantly high academic stress compared to Chinese and Indian students; while Chinese students have higher stress compared to Malay students for financial stress.^6^ Stress is one of the important factors that affect academic performance of the students.^7^

Medical students are exposed to regular pressure with overwork of academic burden and examination that brings various changes in their daily routine such as lack of sleep, irregular diet, smoking and substance abuse in order to cope with stress.^1^^,^^8^ Coping is a way that a person reacts or responds towards stressors. Failing to cope with stress effectively causes deterioration of academic and professional performances and increases the psychological distress.^1^^,^^9^ Coping strategies, if used effectively, may prevent the unwanted consequences of stressful situation.^1^^,^^4^

The objective of this study was to determine the prevalence of stress and coping strategies among UKM first and third year medical students aimed to propose remedial measures.

## METHODS

This was an observational study conducted among first and third year UKM medical students of academic session 2013/2014. Sample size comprised of 234 students, 99 of whom were from first year and 132 from third year, selected using stratified random sampling technique. Data was collected using a stress arousal check list^10^ and a coping inventory for stressful situations.^11^ Stress dimensions in the check list referred to the subjective perceptions of the current situation as unpleasant in response to the external environment through some positive and negative adjectives such as tense, relaxed, apprehensive and worried. Arousal means someone is having a generalized state of increased somatic or autonomic ongoing physiological activities, measured through similar adjectives such as energetic, drowsy, stimulated and activated.

There were 30 adjectives of which 18 were for stress and 12 were for arousal identification. The stress-positive adjectives were measured by sign “++” and “+” scored as “1” while stress-negative adjectives were measured by sign “?” and “–“ scored as “0”. The coping inventory used 48 items, categorized under task, emotion, and avoidance strategies which were measured using 5-point Likert-scale. The questionnaire also contained socio-demographic data of the respondents. Written consent was obtained from the participants before administering the questionnaire, and participants were assured that participation would not affect them anyway. Data was analyzed using SPSS version 20.

## RESULTS

**Which statistica test was used should be added.**

Out of 234 participants 231 responded giving a response rate of 99%. Table-I shows the demographic profile of the respondents which showed that, an average of 66.67% of the respondents were female, 64.4% were Malays and 96.75% were resided in the hostel and 97% were single. Most of the respondents were motivated to study medicine as their own interest.

Table-II showed the prevalence of stress and arousal among the respondents where an average of 48.6% were stressed and 51.4% were aroused. Females and Malays were more stressed compared to male and non Malays while males and non Malays were more aroused than female and Malays.

Table-III shows there was no significant differences of stress and arousal scores between males and females both in first and third year medical students; however, significant differences of stress scores between Malays and Non Malays both in 1^st^ year (p=0.04) and in 3^rd^ year (p=0.01) students were observed with insignificant differences in arousal.

Table-IV shows the distribution of coping strategies among the respondents. The most commonly used coping strategies among the respondents were task oriented followed by avoidance and then emotion oriented coping with insignificant differences in regards to study- year, gender and ethnicity.

**Table-I:** Demographic profile of the respondents, n=231

**Variables**		**First Year, n=99**		**Third Year, n=132**
		**n (%)**		**n (%)**
Gender	Male	33 (33.3)		44 (33.3)
	Female	66 (66.7)		88 (66.7)
Ethnicity	Malay	69 (69.7)		78 (59.1)
	Chinese	15 (15.2)		49 (37.1)
	Indian	15 (15.2)		5 (3.8)
Residency	Hostel	97 (98)	126 (95.5)	
	Parental Home	2 (2.0)	0 (0.0)	
	Rented Accommodation	0 (0.0)	6 (5.5)	
Marital Status	Single	98 (99)		126 (95.5)
	Married	1 (1)		6 (4.5)
Motivation to Study Medicine	Own Interest	83 (83.8)		81 (61.4)
	Family Influence	8 (8.1)		19 (14.4)
	Randomly Choose	8 (8.1)		32 (24.2)

**Table-II:** Distributions of prevalence of stress and arousal among the respondents

**Variables**		**Stress prevalence n (%)**	**Arousal prevalence n (%)**
Year of study	First Year (n=99)	49 (49.5)	50 (50.5)
	Third Year (n=132)	64 (47.7)	69 (52.3)
Gender	Male (n=77)	35 (45.5)	42 (54.5)
	Female (n=154)	77 (50)	77 (50)
Ethnicity	Malay (n=147)	76 (51)	72 (49)
	Non Malay (n=84)	37 (44)	47 (56)
Total (n=231)		112 (48.5)	119 (51.5)

**Table-III:** Distribution of stress and arousal level in terms of their mean score ± SD among the respondents

**Variables**		**First Year**				**Third Year**			
		**Stress** **score ± SD**	***p*** ** value**	**Arousal score ± SD**	***p*** ** value**	**Stress** **score ± SD**	***p*** ** value**	**Arousal score ± SD**	***p*** ** value**
Gender	Male	4.55±2.75	0.73	5.30±2.02	0.70	6.41±3.69	0.41	7.43+2.14	0.24
	Female	4.74±2.53		5.14±1.94		7.00±4.12		6.94±2.46	
Ethnicity	Malay	5.00±2.72	*0.04	5.19±2.10	0.98	7.43+2.14	*0.01	7.37±2.33	0.12
	Non Malay	3.93±2.13		5.20±1.63		5.76±3.31		6.72±2.38	

**Table-IV:** Distribution of coping strategies among the respondents

**Variables**	**Coping strategies**	**First year** **Mean score ± SD**	***p*** ** value**	**Third year** **Mean score ± SD**	***p*** ** value**
Gender	Task-oriented				
	Male	63.21±8.44	0.65	58.66±9.01	0.30
	Female	62.38±8.81		60.47±9.97	
	Avoidance-oriented				
	Male	51.12±11.89	0.39	51.14±9.89	0.37
	Female	53.30±11.75		52.81±10.40	
	Emotion-oriented				
	Male	44.09±11.38	0.58	42.27±10.21	0.85
	Female	42.76±11.33		42.61±9.20	
Ethnicity	Task-oriented				
	Malay	62.38±9.25		59.83±10.45	
	Chinese	61.60±5.57	0.50	59.29±8.52	0.34
	Indian	65.00±8.38		66.00±5.88	
	Avoidance-oriented				
	Malay	51.97±12.16		52.18±11.17	
	Chinese	53.53±9.39	0.73	52.24±8.83	0.968
	Indian	54.40±12.65		53.40±9.29	
	Emotion-oriented				
	Malay	44.00±11.11		42.59±10.31	
	Chinese	39.80±10.69	0.43	41.55±8.14	0.14
	Indian	52.93±12.86		50.40±5.73	

## DISCUSSION

People suffer from stress for many reasons;^12^ a student can be stressed due to educational, financial, health related, loss of close family member or friend, relationship problems and so on.^7^ This study focused on medical students during transitional periods; notably first year where students have to adjust a different environment in the university and third year where students have to adopt a hospital environment where they undergo clinical examinations on patients. The overall prevalence rate of stress in the present study was 49% which was higher compared to 43% in earlier study.^4^ Medical students’ stress prevalence ranged from 30% to 50%,^3^^,^^13^ which is higher from other disciplines.^14^^,^^15^ Although 89% respondents in first year and 61% in third year were motivated to study medicine because of their own interest (Table-I), the stress prevalence was still high.

Stress prevalence in first year students was higher compared to third year while prevalence of arousal was more in third year (Table-II). High prevalence of stress in first year may be due to the introduction of taking more responsibility for their learning and a shift from the traditional teacher-cantered teaching methodology^16^ to self-directed student-centred teaching methodology. Increased prevalence of arousal in third year students may be due to some sorts of adaptability by them. However, the result differs from a previous study done at Universiti Sains Malaysia students where stress-prevalence in third year found higher compared to first year students.^17^ Stress and arousal prevalence were higher in female students compared to males (Table-II), which is consistent with findings of Johari *et al*.^4^ and Abdulghani *et al*.^18^ Malay students have higher prevalence of stress compared to non-Malays which were also consistent with the findings of Johari *et al*.,^4^ but Malays were found with less prevalence of arousal.

Based on level or mean stress score, female students were also shown more mean stressed scores than males but male students were more aroused than females with insignificant differences (Table-III). It may be due to their higher self expectation, feeling less competent, and tendency to over-report symptoms.^19^ Students were stressed both in first and third year, but third year students were more stressed than first year. Supe^20^ showed that stress were high in third year medical students than first year, and this supports our study findings. Malay students were significantly more stressed than non-Malays both in first (p=0.04) and third (p=0.01) year (Table-III) and this findings is consistent with findings of Johari *et al.*^4^ It is however different from the findings of Sherina *et al.*^3^ where Indians were more stressed followed by Malays, Chinese and others.

It is the persons’ ability to cope with day to day challenges which determine whether he/she will be stressed or not and at what level.^6^ Most common coping strategies used by the respondents was task oriented followed by avoidance and emotion (Table-IV). Task-oriented coping is describes as purposeful task effort aimed at solving the problem, or attempts to alter the situation while avoidance-oriented coping describes activities and cognitive changes aims at avoiding the stressful situation. This finding is consistent with findings of Wan Salwina *et al.*^21^ Students used a combination of both emotion and problem oriented coping. Emotion oriented coping describes turning to religion, positive reinterpretation, acceptance and seeking of socio-emotional support and problem-oriented coping describes active coping and planning; but most preferred emotion-oriented coping strategies.^22^

Medical students at UKM found to be highly resourceful to manage stress through exposure of various activities such as decision making in a tough situation, reflective writings, managing diversity, breaking bad news, interfaith discussion, spiritual development and so many other personal and professional development activities over the whole period of five year curriculum.^7^ Even then, stress prevalence and its level among UKM medical students in the present study is higher. Stressors need to be identified and managed effectively. Educational environment exerts an unintentional negative effect with increase stress among medical students.^1^^,^^23^ Students needs a non-threatening supportive learning environment to get relief from academic and examination related stress^1^ Time is changing and teachers’ role is changing from being a knowledge dispenser to a creative designer and facilitator of learning experiences.^24^ Effective educational program with joint collaborative efforts are important.^25^ Educators must address these issues, and ensure a supportive learning environment aimed to develop confidence and better adjustment of students in classroom, group and in society.

## CONCLUSION

The overall prevalence of stress is high among UKM medical students. Female and Malay students are more stressed. Main coping strategies used by respondents are task oriented followed by avoidance and emotion. To get relieve of academic related stress from students, teachers need to ensure a stress free supportive learning environment and act as a creative designer and facilitator of learning experiences rather than act as information provider. Further research should aim to identify the stressors, specific ways to cope and effectiveness of coping among the students aimed to propose appropriate remedial actions. 
